# Where Are All the *Mycobacterium avium* Subspecies *paratuberculosis* in Patients with Crohn's Disease?

**DOI:** 10.1371/journal.ppat.1000234

**Published:** 2009-03-27

**Authors:** Ellen S. Pierce

**Affiliations:** Spokane Valley, Washington, United States of America; University of British Columbia, Canada

## Abstract

*Mycobacterium avium* subspecies *paratuberculosis* (MAP) causes a chronic granulomatous inflammation of the intestines, Johne's disease, in dairy cows and every other species of mammal in which it has been identified. MAP has been identified in the mucosal layer and deeper bowel wall in patients with Crohn's disease by methods other than light microscopy, and by direct visualization in small numbers by light microscopy. MAP has not been accepted as the cause of Crohn's disease in part because it has not been seen under the microscope in large numbers in the intestines of patients with Crohn's disease. An analysis of the literature on the pathology of Crohn's disease and on possible MAP infection in Crohn's patients suggests that MAP might directly infect endothelial cells and adipocytes and cause them to proliferate, causing focal obstruction within already existing vessels (including granuloma formation), the development of new vessels (neoangiogenesis and lymphangiogenesis), and the “creeping fat” of the mesentery that is unique in human pathology to Crohn's disease but also occurs in bovine Johne's disease. Large numbers of MAP might therefore be found in the mesentery attached to segments of intestine affected by Crohn's disease rather than in the bowel wall, the blood and lymphatic vessels running through the mesentery, or the mesenteric fat itself. The walls of fistulas might result from the neoangiogenesis or lymphangiogenesis that occurs in the bowel wall in Crohn's disease and therefore are also possible sites of large numbers of MAP. The direct visualization of large numbers of MAP organisms in the tissues of patients with Crohn's disease will help establish that MAP causes Crohn's disease.

*Mycobacterium avium* subspecies *paratuberculosis* (MAP) causes a chronic granulomatous inflammation of the intestines in dairy cows called Johne's disease. MAP also causes a chronic inflammation of the intestines in beef cattle and in a wide variety of other domestic and wild ruminants as well as other mammals, including nonhuman primates. “MAP is a specific cause of chronic inflammation of the intestines in many different ruminants, including rare species, monogastrics such as dogs and pigs and, so far, four different types of subhuman primates – macaques, baboons, gibbon [sic] and cotton-top tamarins” [1]. MAP has been identified in humans with a chronic granulomatous inflammation of their intestines, Crohn's disease.

The identification of MAP organisms in patients with Crohn's disease has been accomplished by several different techniques. MAP has been cultured from the intestines [2]–[4] and blood [5] of Crohn's patients. In addition, MAP has been identified in the intestines [3], [4], [6]–[12] and blood [5],[11],[13] of Crohn's patients by PCR amplification of the IS900 DNA sequence specific for MAP. IS900 PCR also identified MAP in two cervical lymph nodes of a teenager; 5 years later he developed Crohn's disease [14]. Antibodies to MAP antigens have been identified in the blood of Crohn's patients by ELISA [11], [15]–17. DNA in-situ hybridization has permitted direct visualization by light microscopy of small numbers of MAP organisms in Crohn's intestines [12], [18]–[21]. In 2007, in a single study, MAP was directly visualized by light microscopy in small numbers in Crohn's intestines using the traditional Ziehl-Neelsen acid fast stain ([22], Text S1).

While other organisms have also been identified in the intestines of patients with Crohn's disease [23], no other putative pathogenic organism causes a chronic granulomatous inflammation of the intestines in every other species in which it is present. “For MAP in this situation not to contribute to pathogenesis and merely to have a bystander role, it would be necessary to accept that despite its specific ability to cause chronic inflammation of the intestine in so many animals, including primates, it is somehow harmless to man” [24].

Some argue that MAP has met both Koch's postulates ([25], Text S1) and Relman's criteria [26] for microbial causation of Crohn's disease. MAP is present in pasteurized milk [27], infant formula made from pasteurized milk [28], breast milk from women with Crohn's disease [29], surface water [30]–[32], soil [30], cow manure “lagoons” that can leach into surface water [33], cow manure in both solid and liquid forms that is applied as fertilizer to agricultural land [33], and municipal tap water [25],[34], providing multiple routes of transmission to humans.

Yet, MAP is still not accepted as the cause of Crohn's disease. The identification of MAP by methods other than direct visualization by light microscopy, and the identification of MAP in small numbers by light microscopy, has not constituted convincing evidence of causation to the medical community at large. When MAP is not completely ignored as a cause of Crohn's disease [35], it is dismissed in part because of this lack of visualization of large numbers of the organism ([36], Text S1):

Even advocates of the theory that MAP causes Crohn's disease concede that infection, if present, consists of a low bacterial load …. [37]

Acid fast bacilli have not been found in the tissues of patients with Crohn's disease, leading investigators to speculate that these organisms are not present (or) the bacterial load is very low …. [38]

Is it possible that large numbers of MAP are actually present in some of the histologic (microscopic) lesions of patients with Crohn's disease? Current MAP researchers argue that MAP infection in humans exists only in a paucibacillary form, i.e., only small numbers of organisms are present in the histologic lesions [36]. Instead, the lack of direct visualization of large numbers of the organism in histologic sections of Crohn's tissues might be due to the fact that the organism has not been looked for in locations where it might be present in large numbers. Where might MAP be present in large numbers in patients with Crohn's disease?

An analysis of the literature on the pathology of Crohn's disease and on possible MAP infection in Crohn's patients leads to the following conclusions. First, that some patients with Crohn's disease might have pluribacillary disease, i.e., they might have large numbers of MAP organisms in some of their histologic lesions. Second, that in addition to macrophages, MAP might directly infect and replicate within two other cell types. MAP might infect and replicate within endothelial cells, both the endothelial cells lining lymph vessels and the endothelial cells lining blood vessels, and MAP might infect and replicate within adipocytes or fat cells. Third, MAP organisms might therefore be present in large numbers in the following locations in patients with Crohn's disease:

1. The blood vessels, lymph vessels (lymphatics), and lymph nodes in the mesentery of affected bowel wall segments.
2. The mesentery itself, i.e., the adipocytes that fill the mesentery.
3. The walls of fistulas.

## Do Some Patients with Crohn's Disease Have Pluribacillary MAP Infection?

The two major mycobacterial diseases of humans are tuberculosis and leprosy. The presence of both *Mycobacterium tuberculosis* and *Mycobacterium leprae* varies within histologic lesions, from pluribacillary or multibacillary (having large numbers of organisms within histologic lesions) to paucimicrobial or paucibacillary (having small numbers of organisms within histologic lesions or none at all [39],[40]). If Crohn's disease is, like *M. tuberculosis* and *M. leprae*, caused by a mycobacterium [25], and if some humans with other mycobacterial diseases have large numbers of the causative organism in some of their histologic lesions, isn't it possible that some patients with Crohn's disease might also have large numbers of MAP in some of their histologic lesions, if only the right lesions were examined?

The idea that some patients with Crohn's disease might have large numbers of MAP organisms in their tissues is supported by correlation with both human *M. leprae* infection and bovine MAP infection. The classic lepromatous form of leprosy demonstrates large numbers of organisms in the histologic lesions, and antibodies to *M. leprae* antigens in the blood [40]. Cattle with Johne's disease that have “multibacillary lesions tend to have high serum antibody concentrations” [41], and humans with Crohn's disease have antibodies to MAP antigens in their blood. ELISA studies, which detect antibodies to MAP antigens in the blood, demonstrate that anywhere from 23% to almost 90% of Crohn's patients have such antibodies [11], [15]–[17].

MAP is a mycobacterium. Other mycobacterial infections in humans have some histologic lesions that contain large numbers of the causative organism. If Crohn's disease is caused by MAP, then like other mycobacterial species that cause human disease, large numbers of MAP organisms might be present in some of the histologic lesions of Crohn's disease, organisms that because of their presence in large numbers could be easily seen under the microscope (with the 40× objective rather than the 100× oil immersion objective [36]), if only we looked in the right places.

## What Cell Types Might Be Infected by MAP in Crohn's Disease?

What is the fundamental pathologic problem in Crohn's disease? Most researchers argue that Crohn's disease is a problem of the intestinal epithelial cell. Some kind of defect in the intestinal epithelium allows normal (or “commensal”) gut bacteria through the epithelium into the deeper bowel wall, setting up an autoimmune response [35],[42]. A careful reading of the literature on the pathology of Crohn's disease suggests that endothelial cells and adipocytes rather than intestinal epithelial cells might determine the unique pathologic features of Crohn's disease.

The potential role of adipocytes in the pathology of Crohn's disease will be discussed later in this article. The following sections will consider the role of endothelial cells. Endothelial cells line both lymph vessels and blood vessels, forming the intima or inner layer of these vessels along with a basement membrane. Since the term “vascular” is generally used to refer to blood vessels only, endothelial cells lining blood vessels will be referred to as vascular endothelial cells throughout this article, and endothelial cells lining lymph vessels will be referred to as lymphatic endothelial cells.

The literature suggests that both lymphatic endothelial cells and vascular endothelial cells proliferate in Crohn's tissues. The endothelial cells proliferate within already existing vessels, causing obstruction within those vessels (and depending on whether vascular or lymphatic, a lesser or greater propensity for granuloma formation), and they proliferate and migrate, forming the basis of new blood (neoangiogenesis) or lymphatic (lymphangiogenesis) vessels.

## Does a Proliferating Lymphatic Endothelial Cell Explain Some of the Pathologic Changes in Crohn's disease?

In a recent review article [43], Van Kruiningen and Colombel present evidence that Crohn's disease was historically considered to be a chronic lymphangitis. They conclude that since Crohn's disease involves a chronic inflammation of lymphatic vessels, that the endothelium lining these lymphatic vessels is the location where the etiologic agent might be located. They list a variety of bacteria and viruses that are known to directly infect lymphatic endothelium [43].

Van Kruiningen and Colombel believe that the older articles were describing a chronic lymphangitis, an inflammation of the lymphatics, i.e., an infiltration of the lymphatic lumens or walls by lymphocytes. A close reading of the early articles they cite, however, reveals a different process. Instead of a chronic inflammation of the lymphatics, there is a proliferation of lymphatic endothelial cells in Crohn's disease, resulting in focal obstruction, including granuloma formation, and lymphangiectasia, dilation of the vessels on either side of the obstruction.

Hadfield (1939) writes that the “affected germinal center was entirely replaced by proliferating endothelial cells with weakly-staining nuclei … in the midst of the cells constituting these endothelial aggregates it was usually possible to identify a Langhans' giant cell … it became obvious that endothelial proliferation continued in the giant cell system” [44].

**Figure 1:**
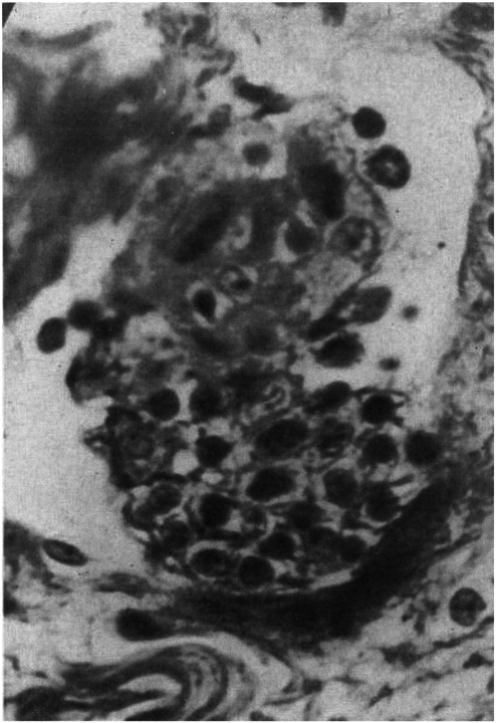
“Ileum in Chronic Cicatrizing Enteritis, Showing Endothelial Cells Proliferating and Blocking a Submucosal Lymphatic. Phosphotungstic Acid – Hematoxylin Stain. ×800” Reads the Original Legend for This Photograph. Note the need for magnification greater than ×400 to accurately identify the cells as endothelial cells. Large numbers of MAP organisms might be found in these proliferating endothelial cells, before they become granulomas. Very kindly reprinted from Am. J. Pathol. 1948, 24: 475–501 with permission from the American Society for Investigative Pathology [45].

Warren and Sommers (1948) write:

In the earliest stage, small foci of leukocytes closely resembling the endothelial cells of lymphatics appear in the lacteals of the lamina propria, between the glands and the muscularis mucosae. These endothelial cells change from flat to polygonal, with abundant eosinophil cytoplasm and somewhat prominent hyperchromatic nuclei. Proliferation of these cells continues and finally blocks the lymphatics [Figure 1]. In slightly later stages similar masses of proliferating endothelium obstruct lymphatics in the submucosa and subserosa…The reaction is sharply focal and intervening stretches of the lymphatics are dilated…Once the larger lymphatics become completely blocked deep in the submucosa and subserosa as well as in the lamina propria, eosinophils and then lymphoid cells surround these endothelial masses …. The endothelial cells become more closely massed and tend to coalesce, forming giant cells. [45]

**Figure 2:**
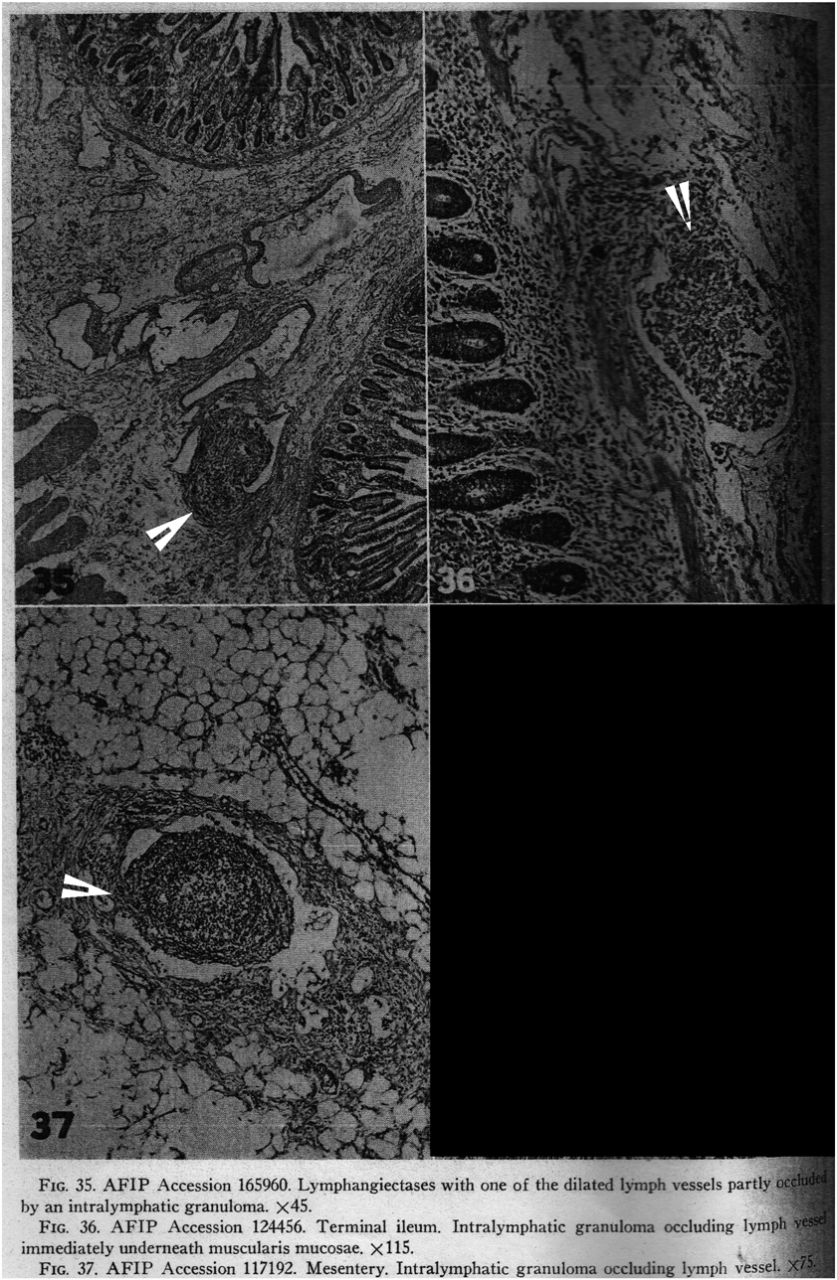
Three Photographs Illustrating Intralymphatic Granulomas. Photograph 35 shows the combination of occlusion by a granuloma and accompanying lymphangiectasis. Photographs 36 and 37 show intralymphatic granulomas. The arrows (added by the author) point to the focal origins of the granulomas in discrete segments of endothelial cells lining the lymphatic vessels. Large numbers of MAP organisms might be found in these endothelial cells, rather than within the fully formed granulomas. Reprinted from Military Surgeon 1951, 109: 463–502, a publication of the Armed Forces Institute of Pathology, with the very kind assistance of Dr. Leslie Sobin [75].

In both of the above descriptions, the swollen and proliferating endothelial cells cause focal occlusions of the lymphatics within which they lie. Shockingly to all current readers, who know granulomas to be collections of modified macrophages, both Warren and Sommers and Hadfield carefully describe the transformation of these proliferating endothelial cells into granulomas (Figure 2). They then describe the secondary ulceration, fissures, and fistulas that are a consequence of the primary process of lymphatic obstruction.

Neither of these older studies describes lymphangiogenesis, the development of new lymphatic vessels from preexisting ones. A recent study, however, describes a markedly increased density of small lymphatic vessels in the submucosa of Crohn's small intestine resection specimens, and in “every layer of the bowel wall, except in the lamina propria,” in colon resection specimens from Crohn's patients [46].

## Does a Proliferating Vascular Endothelial Cell Explain Some of the Pathologic Changes in Crohn's Disease?

The literature investigating the role of bowel wall or mucosal layer blood vessels in the pathogenesis of Crohn's disease provides evidence of three vascular abnormalities in Crohn's tissues:

1. injury to individual vascular endothelial cells, that might represent the initial invasion of those cells by MAP,
2. diffuse proliferation of vascular endothelial cells within already existing blood vessels, causing occlusion of those vessels but only occasional granuloma formation, and
3. angiogenesis, also called neoangiogenesis or neovascular proliferation, the development of new blood vessels, the result of the proliferation and migration of vascular endothelial cells.

Recently, researchers have argued that Crohn's disease is a disease of the small blood vessels, the so-called microvasculature, of the bowel wall [47]. There seem to be two problems with the microvasculature. The first problem is specific defects in or damage to individual vascular endothelial cells in the mucosal layer of both Crohn's disease and ulcerative colitis intestines. The studies of Binion and colleagues, for example, demonstrate that both Crohn's disease and ulcerative colitis are characterized by specific “acquired defects” of vascular endothelial cells lining post capillary venules [48],[49]. A study by Sankey and colleagues describes three changes in mucosal blood vessels, two of which are found in both Crohn's disease and ulcerative colitis, and a third rare “summit” lesion of the mucosal capillary wall that might be unique to Crohn's disease [50].

The second problem with the mucosal capillaries in both Crohn's disease and ulcerative colitis is an apparent tendency to sprout new blood vessels. In addition to the studies of defects in or damage to individual mucosal layer vascular endothelial cells, a separate literature has developed suggesting that angiogenesis, “the process of new capillary formation from preexisting vasculature in adult tissues” [51], is a component in the development of Crohn's disease and ulcerative colitis [51],[52].

This pathologic angiogenesis is very carefully described in the mucosa of both Crohn's disease and ulcerative colitis. What appears to be unique to Crohn's disease is that this neovascular proliferation is present not only in the mucosal layer but in the deeper bowel wall as well. A study by Wakefield and colleagues describes a “mesenteric granulomatous vasculitis” of intestines affected by Crohn's disease [53]. A careful reading of this study, however, reveals that the blood vessels involved are not inflamed and are not in the mesentery. There is occlusion of blood vessels, by fibrin, with endothelial cell “prominence,” in the muscularis propria. The vascular “injury” consists of “intense neovascularization” in the submucosa and subserosa [53].

The current literature describes neoangiogenesis involving the mucosal layer in both Crohn's disease and ulcerative colitis, and the single study just mentioned suggests the presence of neoangiogenesis in the deeper bowel layers in Crohn's disease. In contrast, older literature describes occlusive changes in the bowel wall in Crohn's disease, including what is specifically described as intimal proliferation, i.e., the proliferation of the endothelial cells that constitute the intima. These older studies of the vasculature in Crohn's disease, like the earlier studies of the lymphatic system, do not emphasize inflammation in the blood vessels but rather “obliterative changes, including intimal proliferation, subintimal fibrosis, medial hypertrophy, medial fibrosis, and adventitial fibrosis, without significant inflammatory cell component” ([54], emphasis added). For example, Van Patter and colleagues (1954) write: “Localized and diffuse changes in both arteries and veins of the bowel wall were encountered. Thickening of the intima, both cellular and acellular, was more prominent in the arteries than in the veins” [55]. They note specifically that “[i]dentical changes in the intima of blood vessels of tuberculous cattle have been reported … to follow intradermal injection of tuberculin” that the accompanying figures describe as a “proliferative endarteritis” ([55], emphasis added). This diffuse, circumferential proliferation of vascular endothelial cells is so thick as to completely occlude the lumen of the blood vessel.

While intimal proliferation is the most common description of the changes within blood vessels, occasional granuloma formation within blood vessels is also noted. Geller and Cohen [54] describe vessels where both diffuse intimal proliferation and granuloma formation occur together. “[I]ntimal-medial hypertrophy was also noted …. These specimens demonstrated granulomatous involvement of a vessel with obvious interruption of the internal elastic lamina.” One of their figures is a “granulomatous inflammation involv[ing] adventitia, media, and proliferated intima” [54]. One of the more recent granuloma studies [56] also mentions the occasional granuloma occluding subserosal arterioles.

**Figure 3:**
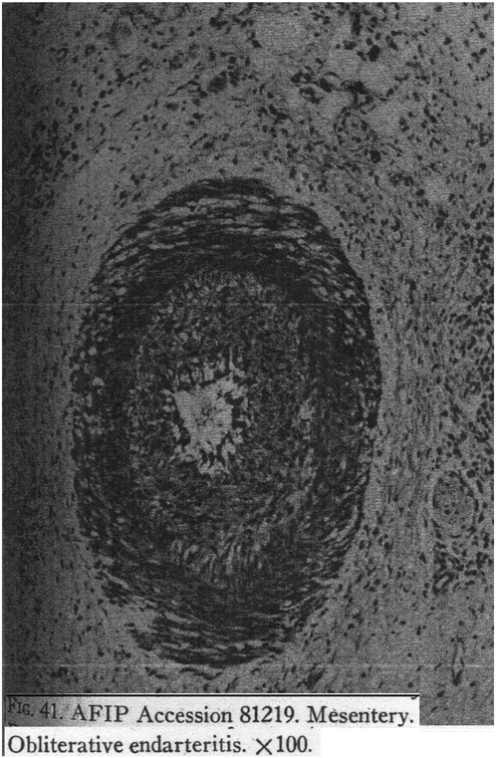
This Photograph of “Obliterative Endarteritis” Illustrates the Diffuse Endothelial Cell Proliferation within Blood Vessels, Causing the Circumferential Widening of the Intima, in Contrast to the Focal Proliferation of Endothelial Cells within Lymphatics, Resulting in Granulomas. Large numbers of MAP organisms might be found within the endothelial cells of the thickened intima. Reprinted from Military Surgeon 1951, 109: 463–502, a publication of the Armed Forces Institute of Pathology, with the very kind assistance of Dr. Leslie Sobin [75].

The literature suggests that the proliferation of lymphatic endothelial cells in Crohn's disease occurs primarily within already existing vessels and is focal or segmental (Figure 2) rather than diffuse or circumferential, causing granuloma formation that obstructs the vessel and lymphangiectasis, and only secondarily causing lymphangiogenesis. In contrast, the proliferation of vascular endothelial cells in Crohn's disease appears equally likely to cause both neoangiogenesis, the development of new blood vessels, and circumferential thickening of the intima within already existing blood vessels (Figure 3), but only occasional granuloma formation.

## What Causes Endothelial Cells to Proliferate in Crohn's Disease?

If proliferating endothelial cells are the basis of the pathologic changes in Crohn's disease, what causes them to proliferate? If MAP causes Crohn's disease, then direct infection of endothelial cells by MAP might be the reason that endothelial cells proliferate in Crohn's disease.

Can MAP directly invade (or “infect”) endothelial cells? MAP in dairy cattle with Johne's disease are engulfed by subepithelial macrophages that then “migrate into local lymphatics, spreading the infection to regional lymph nodes” [57]. It is not known whether MAP can directly infect the endothelial cells lining the lymphatics within which the macrophages travel. “There is little known about where exactly viable MAP can be found in human tissues or, since most pathogenic Mycobacteria are intracellular, in which cells MAP can live and grow in humans. While the site of infection and tissue pathologies of MAP in animals can be assessed at necropsy, there is enough dissimilarity between digestive processes of ruminants and humans that this information may not necessarily inform studies in humans” [58].

Other *Mycobacterium* species, however, are known to infect and replicate within endothelial cells. *Mycobacterium tuberculosis* can infect and replicate within vascular endothelial cells [59], and *Mycobacterium leprae* can infect and replicate within both vascular and lymphatic endothelial cells [60].

Does the invasion of endothelial cells by microorganisms cause them to proliferate? Recent evidence that “indigenous” microbes [61] and particular strains of *E. coli* [62] can stimulate angiogenesis, and *Bartonella* species can both infect endothelial cells and stimulate angiogenesis [63], suggests that angiogenesis or lymphangiogenesis, the neovascular proliferation of endothelial cells, is a possible mechanism whereby MAP establishes chronic infection.

## So Where Are All the MAP in Patients with Crohn's Disease?

If some patients with Crohn's disease might have pluribacillary MAP infection, i.e., if some of their histologic lesions might have large numbers of MAP organisms, and if MAP might be present within endothelial cells lining lymphatic and vascular walls, then two kinds of places where no one has yet looked for them by either direct or indirect methods might have abundant MAP organisms—places where there are large blood vessels or lymphatics and places where there many small blood vessels or lymphatics.

Where are large blood vessels or lymphatics where abundant MAP organisms might be located? The large blood vessels and lymphatics that supply and drain the intestines are in their attached mesentery. These mesenteric blood vessels and lymphatics, located outside of the bowel wall, might have large numbers of MAP organisms.

Where are collections of small lymph or blood vessels where large numbers of MAP organisms might be located? The fistulas of patients with Crohn's disease, whose walls are usually described as being composed of capillaries (i.e., small blood vessels) but might instead be small lymphatic channels as discussed below, are a unique location where large numbers of MAP organisms might be found.

## The Evidence for Lymphatics in the Mesentery as a Possible Site of Large Numbers of MAP Organisms in Crohn's Disease

The evidence is strongest that the endothelial cells lining the walls of mesenteric lymphatics are a possible site of large numbers of MAP organisms in Crohn's tissues. The early histopathologic studies of “regional ileitis” [45], the drainage studies of the lymphatics during surgery [64],[65], and mesenteric lymphatic complications of Crohn's disease [66] all suggest that the mesenteric lymphatics are obstructed adjacent to the segments of intestine affected by Crohn's disease. Additionally, the only animal model that closely matches the features of Crohn's disease was produced by injecting formalin into the mesenteric lymph nodes draining the terminal ileums of pigs, producing obstruction of the mesenteric lymphatics draining from those lymph nodes [67].

One feature that is unique in human pathology to Crohn's disease is “creeping fat” or “fat wrapping,” where the mesenteric fat wraps around the bowel wall of intestinal segments affected by Crohn's disease (see discussion below). Another phenomenon, the development of fistulas (also discussed below), while not completely unique to Crohn's disease, does distinguish Crohn's disease from ulcerative colitis. This porcine model of Crohn's disease is unique to all animal models of bowel inflammation in that the pigs developed fistulas, and while the “wrapping phenomena typical of Crohn's disease was not present in (the) animals, probably due to the short follow-up time,” the mesentery was “oedematous” and “clearly thickened” [67]. No other animal model of Crohn's disease has produced fistulas in the affected animals or produced anything close to the mesenteric creeping fat in Crohn's disease.

In this porcine model, the destroyed lymph nodes were equivalent to the focal obstruction of the lymphatic vessels in Crohn's disease caused by proliferating endothelial cells. There was lymphangiectasia in the bowel wall and in the mesentery on either side of the obstructed lymph nodes, as there is lymphangiectasia on either side of the focal proliferations of endothelial cells in the lymphatics in Crohn's tissues. There were granuloma-like formations (although tubular rather than round) in the subserosa and mesentery in the pigs. This study reproduced every pathologic feature of Crohn's disease [67].

Other studies show that the obstruction of mesenteric lymphatics in Crohn's disease correlates with the pathologic alterations in the mucosa and deeper bowel wall. Van Kruiningen and Colombel [43] describe a series of studies by Tonelli [68] that make the same argument for mesenteric lymphatics that Wakefield and colleagues make for mesenteric blood vessels as described below—that the mucosal ulcers along the mesenteric border in Crohn's disease correlate with the lymphatic drainage into the mesentery. In addition, according to Van Kruiningen and Colombel [43], Tonelli argues that the differing lengths of the lymphatics draining the jejunum and ileum are correlated with the differing lengths of the linear ulcers in Crohn's disease. Shorter vasa recta lymphatics drain the jejunum; the linear ulcers in the jejunum are short. Longer vasa recta lymphatics drain the ileum; the linear ulcers in the ileum are long. This contrasts with the vasa recta arterial supply to the intestines, where the vasa recta arteries supplying the jejunum are long, and those supplying the ileum are short.

As discussed above, the older literature strongly suggests that the lymphatics are the primary site of granuloma formation and that this will be the case in the mesenteric lymphatics as it is in the bowel wall lymphatics. The literature also suggests that the lymphatic vessels in the mesentery respond to obstruction by lymphangiectasia rather than lymphangiogenesis. The literature to date does not support the idea of a large vessel lymphangiogenesis in the mesentery. Instead, lymphatic obstruction and lymphangiectasia are the probable pathologic processes in the mesenteric lymphatics.

## The Evidence for Blood Vessels in the Mesentery as a Possible Site of Large Numbers of MAP Organisms in Crohn's Disease

As described above, there is both occlusion of already existing blood vessels and neoangiogenesis in the bowel wall in Crohn's disease. Is there evidence of both occlusion of already existing blood vessels and neoangiogenesis in the mesentery in Crohn's disease?

There is evidence that ischemia in general (constriction or occlusion of blood vessels) is involved in the pathogenesis of Crohn's disease. For example, smoking, which increases the risk of ischemic events in a variety of vascular beds, increases the risk of Crohn's disease and worsens its severity and course in already affected patients [69],[70]. The differential diagnosis of Crohn's disease includes ischemic injury of the mesenteric arteries because ischemic occlusion of the mesenteric arteries is known to produce a pattern of injury with clearly defined margins, just as Crohn's disease is characterized by discrete segments of diseased bowel with intervening segments of normal intestine [71]. On the other hand, patients with bleeding disorders such as hemophilia and Von Willebrand disease have a decreased risk of developing Crohn's disease [47]. There are case studies of treating Crohn's disease by the administration of heparin [47].

The vasa recta arteries in the mesentery just outside the bowel wall are the most likely blood vessels in the mesentery to find abundant MAP organisms. These vasa recta arteries begin in the mesentery, as branches off of the terminal arcades. They enter the bowel wall in the subserosa, either as “short” branches off of the “long” vasa recta on the mesenteric side of the bowel wall, or as continuations of the long vasa recta that course around the subserosa and enter the muscularis propria along the antimesenteric side of the bowel wall [72]. They are the most likely place to find abundant MAP organisms because occlusion of these specific arteries would be most likely to produce the sharply demarcated mucosal lesions of Crohn's disease.

As described above, the linear ulcers in Crohn's disease lie along the mesenteric attachment, i.e., where the mesentery first connects with the bowel wall, whether these ulcers are in the jejunum, ileum, or colon. In animal models [73],[74] and postmortem examination of normal bowel anatomy [72], Wakefield and colleagues demonstrate that the short branches of the vasa recta of the jejunum, ileum, and colon are all end arteries, i.e., they did not anastomose with other vessels in the submucosal plexus once they reach the submucosa, whereas the long vasa recta entering the bowel on the antimesenteric side do anastomose with other vessels once they reach the submucosa. They conclude that occlusion, in the mesentery, of the mesenteric side short vasa recta in patients with Crohn's disease would produce the mesenteric side and longitudinal (parallel to the mesentery) mucosal ulcers characteristic of Crohn's disease [72].

As just described, there is evidence that, if the mesenteric vasa recta arteries in Crohn's disease were occluded, then this occlusion would likely cause the transmural ulcers seen in Crohn's disease. But has anyone looked at the arteries in the mesentery of Crohn's intestines under the light microscope? Only older studies have included examination of mesenteric blood vessels in Crohn's patients, and these older studies only mention the mesenteric blood vessels in passing. Rappaport and coworkers (1951) [75] state: “Vascular changes such as chronic phlebitis and obliterating endarteritis [Figure 3] were more prominent in the mesentery and the serosa than in any of the other coats.” Knutson and coworkers (1968) [76] described their obliterative vascular changes in fully developed Crohn's disease as being “numerous both in the submucosa and periintestinal fat.”

There is slight histologic evidence and similarities to other ischemic conditions, suggesting that mesenteric vasa recta occlusion occurs in Crohn's disease. Is there evidence of mesenteric angiogenesis in Crohn's disease?

A series of older angiographic studies show a specific pattern of what appears to be a proliferation of large blood vessels in the mesentery of Crohn's disease–affected intestines [77]–[79]. These studies show that the density of both long and short vasa recta are increased to the segments of intestine affected by Crohn's disease, whereas the vasa recta density is normal to the non-affected intestinal segments in Crohn's patients. Brahme and Lindström (1970) [77] write: “The mesenteric arteries were not noticeably widened, but the number of arteriae rectae supplying the involved area appeared increased …. In small-bowel lesions about 1.2–2.0 such arteries originated per centimeter, which was more than twice the frequency found in normal specimens.” Kalima and colleagues (1975) [78] write: “The long vasa recta branched from the marginal arcades as in the normal ileum, but the distances between these arteries as they ran in parallel towards the antimesenteric border of the gut were much narrower than in normal ileum. So the number of long vasa recta was markedly (3–5 times) increased per unit length of ileum.” Brahme and Hildell (1976) [79] write: “The arteriae rectae longae … were numerically increased in 74 of 115 lesions of the small bowel (64%) and in 28 of 55 colonic lesions (51%).”

The literature suggests that there is both occlusion of already existing vasa recta arteries and neoangiogenesis of the vasa recta arteries in the mesentery attached to segments of intestine affected by Crohn's disease, and that the endothelial cells constituting the intima of these occluded and proliferated blood vessels might contain large numbers of MAP organisms.

## The Evidence for Fistulas in Crohn's Patients as a Possible Site of Large Numbers of MAP Organisms

Fistulas are tracts or communications between anatomically distinct sites that do not normally communicate with each other. The fistulas in Crohn's disease begin in the bowel and end in other sections of the bowel (entero-enteric), in other organs (e.g., rectovaginal), or on the skin surface (entero-cutaneous).

The walls of fistulas are always described as being composed of “granulation tissue” [80],[81]. What is granulation tissue but a mass of capillaries, or small blood vessels? Granulation tissue is part of how the body responds to wounds. Granulation tissue is physiologic angiogenesis [71].

In Crohn's fistulas, however, this granulation tissue, this mass of small blood vessels, is usually permanent [81]. Is the “granulation tissue” in fistulas actually neoangiogenesis, the result of the neovascular proliferation of endothelial cells? Neoangiogenesis affects all of the layers of the bowel wall in Crohn's disease and, as just discussed, might be present in the mesentery as well. Are the small capillaries that compose the walls of fistulas a result of the process of neoangiogenesis in Crohn's disease? The new vessels appear to begin in the mucosa and track along the course of the original vessels into and through the bowel wall.

The known association between various cancers and angiogenesis [82] supports the idea that fistulas are the result of a process of neoangiogenesis. Cancers are a known cause of fistula formation. Are the fistulas caused by cancer also a result of the process of neoangiogenesis?

As mentioned above, lymphangiogenesis has recently been described in the bowel wall in Crohn's patients [46]. It is possible that the walls of fistulas are actually composed of small lymphatic vessels rather than small blood vessels. The same process of formation of these small lymphatics in the mucosa, and tracking along the course of the original lymphatics into and through the bowel wall, could also cause fistula formation.

The characteristic fistulas of Crohn's disease might be caused by the proliferation and migration of endothelial cells, resulting in neoangiogenesis or lymphangiogenesis, that as discussed previously have been observed in the mucosa, deeper bowel wall, and mesentery in Crohn's patients. Since these fistulas are composed of small vessels lined by endothelial cells, whether of lymphatic or vascular origin, fistulas might be a site of large numbers of MAP organisms in patients with Crohn's disease.

## Where Else Are All the MAP in Patients with Crohn's Disease? The Evidence for the Mesenteric Fat as a Possible Site of Large Numbers of MAP Organisms

As mentioned above, “creeping fat” is the phenomenon whereby the mesenteric fat attached to segments of intestine affected by Crohn's disease thickens, stiffens, and wraps around the bowel wall [83]. Creeping fat is absolutely unique in human pathology to Crohn's disease [84]. There is no other disease in humans where the mesenteric fat wraps around the bowel wall. Creeping fat is, however, present in dairy cows with Johne's disease [85].

Despite the singularity of mesenteric creeping fat in human pathology, there is a paucity of literature devoted to the subject [83]. Two lines of evidence, however, suggest that the mesenteric fat attached to Crohn's affected segments of intestine is a possible site of large numbers of MAP organisms.

The first line of evidence is the similarity of adipocytes to macrophages [86]. This similarity extends to the ability of adipocytes to be infected by microorganisms, including the parasite *Trypanasoma cruzi* [87], several viruses [87], and *M. tuberculosis* [88]. In particular, the adipocytes in the mesentery “might be a main reservoir of bacteria” [83]. MAP is known to infect macrophages [57]. Do the similarities between macrophages and adipocytes extend to the ease with which MAP can invade them?

The second line of evidence is the apparent proliferation of adipocytes in the creeping fat. Creeping fat is usually described as a hypertrophy of the mesenteric fat. But while there is a hypertrophy, an increase in size, of the mesentery as a whole, there is actually a hyperplasia, an increase in number, of the individual adipocytes that comprise the mesentery [83],[89]. This hyperplasia of adipocytes might also be described as a proliferation of the adipocytes, similar to the proliferation of endothelial cells that is present in the lymphatics and vasculature in Crohn's intestines. Desruisseaux and colleagues note specifically that “with certain types of adenoviruses, infection with viral particles leads to long-term hyperplasia and hyperproliferation of adipocytes” [87]. Does infection of mesenteric adipocytes by MAP cause them to proliferate, causing the creeping fat that is absolutely unique among human diseases to Crohn's disease, but shared by dairy cows with Johne's disease?

## Why Haven't Large Numbers of MAP Organisms Already Been Found in the Mesentery—in the Lymph Nodes?

One place in the mesentery has been investigated for MAP: the lymph nodes. The earliest descriptions of Crohn's disease carefully noted that the lymph nodes in the mesentery are often enlarged and, irrespective of their size, often contain the same type of giant cell granulomas found in the bowel wall [90],[91]. The mesenteric lymph nodes from Crohn's patients were in the early days carefully examined for *Mycobacterium tuberculosis*, not MAP, and the lack of identification of *M. tuberculosis* in the Crohn's nodes was one of the characteristics that distinguished Crohn's disease from ileocecal tuberculosis.

As detailed by Chiodini, attempts were made to culture mycobacteria other than tuberculosis from Crohn's tissues, including mesenteric lymph nodes, beginning in the late 1970s [92]. In the modern era, a single mesenteric lymph node has been investigated for the presence of, and found to contain, MAP [93]. Why then haven't large numbers of MAP been found in the mesenteric lymph nodes of patients with Crohn's disease?

There are two possible reasons that large numbers of MAP organisms have not been found in the mesenteric lymph nodes. The first reason is that no one has looked very hard. Surprisingly, with the exceptions just noted, there have been no studies investigating mesenteric lymph nodes from patients with Crohn's disease for the presence of MAP by the wide variety of direct and indirect methods currently available [94].

The second possible reason that large numbers of MAP organisms have not been found in the mesenteric lymph nodes is that the wrong nodes might have examined. Only lymph nodes containing granulomas have been examined, and granulomas might not be the site of large numbers of MAP organisms.

## Why the Granulomas in Crohn's Tissues Might Not Be the Site of Large Numbers of MAP Organisms, and Where Exactly Large Numbers Might Instead Be Found

Granulomas are usually described as a reaction to foreign antigen, with the antigen, whether microbiologic or otherwise, being found within the granuloma. Since the etiologic agent of Crohn's disease is assumed to be within the granuloma, investigators have focused their attention on determining exactly where the granulomas are in the bowel wall in Crohn's intestines [56],[95],[96], and attempting, rarely successfully, to identify small numbers of MAP from the granulomas in the bowel wall [97].

If specific etiologic agents cause granulomas, and those specific etiologic agents are usually within the granulomas, why haven't large numbers of MAP organisms been found within Crohn's granulomas? The probable reason is that Crohn's granulomas are formed from no longer proliferating endothelial cells, and large numbers of MAP organisms will probably be present within proliferating endothelial cells instead.

Warren and Sommers write: “Once the larger lymphatics become completely blocked deep in the submucosa and subserosa as well as in the lamina propria, eosinophils and then lymphoid cells surround these endothelial masses in increasing numbers. The endothelial cells become more closely massed and tend to coalesce, forming giant cells. Incomplete stages in this process can be found in which individual endothelial cells are partly fused” [45]. Hadfield is more explicit. He writes: “[I]t became obvious that endothelial proliferation continued in the giant-cell system until it eventually reached a maximal diameter of approximately four to five times that of the germinal centre which it had replaced …. When the giant-cell system has reached this size, cell proliferation apparently ceases” [44]. These descriptions suggest that granulomas, since they are formed from massed, coalescing but no longer proliferating endothelial cells, will not have large numbers of MAP within them.

If large numbers of MAP organisms won't be found within granulomas, where exactly will they be found? In their classic textbook [71], Cotran and colleagues describe a granuloma as follows: “A granuloma … consists of a microscopic aggregation of macrophages that are transformed into epithelium-like cells surrounded by a collar of mononuclear leukocytes, principally lymphocytes and occasionally plasma cells.” There are two components to a granuloma, a collection of epithelium-like cells, and a surrounding collar of mononuclear leukocytes. Large numbers of MAP organisms might be found in the collections of (not macrophages but) endothelial cells, just before they become “transformed into epithelium-like cells” and are surrounded by a “collar of mononuclear phagocytes.”

Regarding the mesenteric lymph nodes, this means that large numbers of MAP organisms might be found in the lymph nodes that were described in the older literature as hyperplastic rather than granulomatous, a hyperplasia of what might be proliferating endothelial cells rather than lymphocytes. Van Patter and colleagues note that in the mesenteric lymph nodes in their Crohn's specimens, “[t]he endothelial cells of the sinusoids had undergone proliferation but the process was more generalized than in the lymphatics. The endothelial cells (phagocytes) occurred in large numbers in the dilated lymph channels” [55]. Mesenteric lymph nodes might have large numbers of MAP organisms during this phase of endothelial cell proliferation, before that proliferation ceases, when the “maximal diameter” of the mass of proliferating endothelial cells is reached, but before the endothelial cells stop proliferating, are transformed into epithelium-like cells, and are surrounded by a collar of mononuclear phagocytes.

A situation similar to the mesenteric lymph nodes might be the case in the mesenteric lymphatics. Large numbers of MAP organisms might be found not in fully formed granulomas but in the masses of proliferating endothelial cells just before they become granulomas (Figure 1). Large numbers of MAP might also be found in the segments of proliferating endothelial cells along the walls of the lymphatics adjacent to granulomas, which appear to be the source of the endothelial cells forming the granulomas (Figure 2).

Since the literature suggests that the mesenteric vasculature won't contain many granulomas, large numbers of MAP organisms might instead be found in the endothelial cells along the walls of the vasa recta arteries that are “increased in number” in the mesentery attached to Crohn's disease–affected segments of intestine, and within the circumferentially proliferating endothelial cells lining (and obstructing) already existing vasa recta arteries in that same mesentery (Figure 3).

## What Are the Other Obstacles to Finding Large Numbers of MAP Organisms in the Tissues of Patients with Crohn's Disease?

In addition to what might be the wasted effort trying to find large numbers of MAP within the granulomas in Crohn's tissues, several other obstacles might prevent the direct visualization of large numbers of MAP organisms in the histologic lesions of patients with Crohn's disease. One major obstacle is that all of the “immunosuppressive” drugs used to treat Crohn's disease might have anti-MAP activity.

The use of steroids in Crohn's disease and other immunosuppressive therapy are considered contraindicated in pulmonary tuberculosis, exacerbating the disease. However, the detrimental effects of immunosuppressive drugs on mycobacterial infections are not as pronounced as believed. Steroids in combination with antimicrobial agents have been used for treatment of leprosy and in tuberculosis and other mycobacterial infections …. Studies in cattle with paratuberculosis have shown that massive corticosteroid administration does not significantly influence the clinical manifestations or outcome of the disease, although it was expected to. Treatment of experimental *M. paratuberculosis* infection in rabbits with methotrexate, a powerful immunosuppressive drug, resulted in clinical improvement even though the bacillary load increased. [92]

In addition to steroids, many of the drugs used to treat Crohn's disease might actually act by inhibiting MAP. Methotrexate [98], 6-mercaptopurine [98], the 5-amino-salicylic acid portion of sulfasalazine [99], cyclosporine A [100], rapamycin [100], and tacrolimus [100] all inhibit the growth of MAP in vitro. Since many current patients with Crohn's disease have been or are undergoing treatment with one of more of these drugs at the time of their surgery, both the modern histopathology of Crohn's disease and the number of MAP organisms within biopsy or resection specimens from Crohn's patients may be quite different from untreated cases. The ideal surgical specimen to test the proposed hypothesis will therefore come from patients whose Crohn's disease is first diagnosed at the time of surgery.

Another obstacle to finding large numbers of MAP organisms in tissues affected by Crohn's disease is the possibility that the MAP organism might be present in a cell wall–deficient or spheroplastic form [101],[102], a form that will not stain with traditional cell wall–based acid fast (Ziehl-Neelsen or rhodamine-auramine) stains. Immunohistochemical stains (directed against various cellular antigens, usually proteins) [103], DNA in-situ hybridization (utilizing a stretch of DNA or RNA against the complementary DNA or RNA in the organism) [12], [18]–[21], or PCR amplification of the IS900 DNA sequence specific for MAP [3]–[5], [6]–[14] might therefore be necessary to identify MAP organisms.

Another obstacle to finding large numbers of MAP organisms in the tissues of patients with Crohn's disease is that large numbers of MAP organisms might not be present. It is of course entirely possible that MAP infection in humans exists only in a paucibacillary form, and so only “rare or no” organisms will be present in the mesentery or fistulas.

Finally, while the author thinks it unlikely that a never before identified organism might be the cause of Crohn's disease, silver staining seems a particularly useful histochemical technique for the visualization of bacteria, and it has never been tested on Crohn's tissues. *Tropheryma whippelii*, the causative agent of Whipple's disease, while usually visualized by PAS (periodic acid Schiff) staining, was seen in “great numbers” in the silver-stained section of a lymph node in the original report [104]. The organism that causes cat scratch disease, *Bartonella henselae*, was not seen under the microscope until researchers finally thought to try the Warthin-Starry silver stain on diseased lymph nodes [105], over 30 years after the disease was first described. While now “easily” seen by the usual H & E stain, *Helicobacter pylori* organisms were visualized by the Warthin-Starry silver stain in Warren and Marshall's first report [106]. The many histochemical stains available for microorganisms [107] do not appear to have been tested in the intestines of patients with Crohn's disease or anywhere else.

## Conclusion

Demonstrating large numbers of MAP organisms in the mesenteric fat, the blood vessels or lymphatics running through the mesenteric fat, the hyperplastic (probably not granulomatous) lymph nodes in the mesenteric fat, or the fistulas from patients with Crohn's disease will help establish that MAP causes Crohn's disease. While fistulectomies are a last resort in patients with Crohn's disease, preliminary research can be performed on more readily available mesenteric fat attached to resected segments of intestine. The author is severely disabled by irreversible complications of Crohn's disease and is unable to test the ideas presented in this article. She reminds microbiologists, pathologists, surgeons, and other scientists and physicians in training that Barry Marshall was an internal medicine fellow when he and pathologist Robin Warren published observations of the direct visualization of *H. pylori* organisms in gastric biopsies [106]. The first step in establishing that a bacterium causes a disease is the consistent direct visualization of the organism by light microscopy “in such numbers, and … in such a manner as to explain the lesions of the disease” [108]. Let's take that first step.

*Dedicated to the memory of Rodger C. Haggitt, M.D.*

## Supporting Information

Text S1 Annotated Bibliography.
(0.15 MB PDF)
