# Supplementary material for: Where Are All the Mycobacterium avium Subspecies paratuberculosis in Patients with Crohn's Disease?
Source: PLoS Pathog. 2009 Mar 27;5(3):e1000234. doi: 10.1371/journal.ppat.1000234 (PMC2654961; doi:10.1371/journal.ppat.1000234)
Supplement: Text S1 — Annotated Bibliography. (0.15 MB PDF) [file ppat.1000234.s001.pdf]

Where Are All the *Mycobacterium avium* Subspecies *paratuberculosis* in Patients with Crohn's Disease?

Ellen S. Pierce
ellenpiercemd@gmail.com

Citation: Pierce ES (2009) Where Are All the *Mycobacterium avium* Subspecies *paratuberculosis* in Patients with Crohn's disease? PLoS Pathog 5(3): 1000234. doi:10:1371/journal.ppat.1000234

Annotated Bibliography (Found at doi:10.1371/journal.ppat.1000234.s001)

1. Hermon-Taylor J, Bull TJ, Sheridan JM, Cheng J, Stellakis ML, et al. (2000) Causation of Crohn's disease by Mycobacterium avium subspecies paratuberculosis. Can J Gastroenterol 14: 521-539.

2. Schwartz D, Shafran I, Romero C, Piromalli C, Biggerstaff J, et al. (2000) Use of short-term culture for identification of Mycobacterium avium subsp. paratuberculosis in tissue from Crohn's disease patients. Clin Microbiol Infect 6: 303-307.
   MAP was cultured from 6/7 ((86% of) full thickness samples of bowel wall (86%) and 4/20 (20% of) the mucosal biopsies.

3. Bull TJ, McMinn EJ, Sidi-Boumedine K, Skull A, Durkin D, et al. (2003) Detection and verification of Mycobacterium avium subsp. paratuberculosis in fresh ileocolonic mucosal biopsy specimens from individuals with and without Crohn's disease. J Clin Microbiol 41: 2915-2923.
   MAP was identified by PCR in 92% of the tested Crohn's samples. 42% of the Crohn's samples were culture positive if the cultures were incubated from 14 to 88 weeks, and 60% of the Crohn's samples were culture positive if the cultures were incubated over 38 weeks.

4. Sechi LA, Scanu AM, Molicotti P, Cannas S, Mura M, et al. (2005) Detection and Isolation of Mycobacterium avium subspecies paratuberculosis from intestinal mucosal biopsies of patients with and without Crohn's disease in Sardinia. Am J Gastroenterol 100: 1529-1536.
   MAP was identified by IS900 PCR in 83.3% of the Crohn's "fresh intestinal mucosal biopsies" and by culture in 63.3 % of the biopsies.

5. Naser SA, Ghobrial G, Romero C, Valentine JF (2004) Culture of Mycobacterium avium subspecies paratuberculosis from the blood of patients with Crohn's disease. Lancet 2004 364: 1039-1044.
   This study used nested PCR to identify MAP in both uncultured buffy coats and cell culture pellets from MGIT tubes. MAP DNA was identified in the uncultured buffy coats in 13/28 (46% of) Crohn's patients, 4/9 (45% of) ulcerative colitis patients, and 3/15 (20% of) nonIBD controls. MAP DNA was identified in the cell culture pellets in "50% (14/28) of the Crohn's patients, 22% (2/9) of the ulcerative colitis patients, and none of the individuals without inflammatory bowel disease."

6. Murray A, Oliaro J, Schlup MM, Chadwick VS (1995) Mycobacterium paratuberculosis and inflammatory bowel disease: frequency distribution in serial colonoscopic biopsies using the polymerase chain reaction. Microbios 83: 217-228.

MAP was identified by IS900 PCR in 22% of the Crohn's biopsies and 13% of the ulcerative colitis biopsies

7. Autschbach F, Eisold S, Hinz U, Zinser S, Linnebacher M, et al. (2005) High prevalence of Mycobacterium avium subspecies paratuberculosis IS900 DNA in gut tissues from individuals with Crohn's disease. Gut 54: 944-949.
   MAP was identified by IS900 PCR in 52% of the Crohn's "surgically resected" tissue samples, and in 2% of the ulcerative colitis samples.

8. Sanderson JD, Moss MT, Tizard ML, Hermon-Taylor J (1992) Mycobacterium paratuberculosis DNA in Crohn's disease tissue. Gut 33: 890-896.
   MAP was identified by IS900 PCR in 65% of the Crohn's "full thickness samples of intestine" and in 4.3% of the ulcerative colitis samples.

9. Dell'Isola B, Poyart C, Goulet O, Mougenot JF, Sadoun-Journo E, et al. (1994) Detection of Mycobacterium paratuberculosis by polymerase chain reaction in children with Crohn's disease. J Infect Dis 169: 449-451.
   Both biopsies and resection samples were tested, but not reported separately. MAP was identified by IS900 PCR in 72% of the Crohn's samples and in 20% of the ulcerative colitis samples.

10. Mishina D, Katsel P, Brown ST, Gilberts EC, Greenstein RJ (1996) On the etiology of Crohn disease. Proc Natl Acad Sci U S A 93: 9816-9820.
    This study utilizing RT-PCR (RNA is first transcribed into its complementary DNA sequence before PCR on that cDNA is performed) found MAP RNA in 8/8 Crohn's biopsies and 2/2 ulcerative colitis biopsies.

11. Feller M, Huwiler K, Stephan R, Altpeter E, Shang A, et al. (2007) Mycobacterium avium subspecies paratuberculosis and Crohn's disease: a systematic review and meta-analysis. Lancet Infect Dis 2007: 7: 607-13.
    This study analyzed 18 case control studies using PCR in either tissue samples or blood, and 10 case control studies using ELISA tests for antibodies to MAP in blood. The pooled odds ratio from the PCR studies was 7.01, ie, patients with Crohn's disease were 7 times more likely to have MAP in their tissues or blood than nonIBD controls. The pooled odds ratio from the ELISA studies was 1.72; patients with Crohn's disease were almost twice as likely to have antibodies to MAP in their blood as nonIBD controls. The authors concluded that "(t)he association of MAP with Crohn's disease seems to be specific, but its role in the etiology of Crohn's disease remains to be defined."

12. Abubakar I, Myhill D, Aliyu SH, Hunter PR (2008) Detection of Mycobacterium avium subspecies paratuberculosis from patients with Crohn's disease using nucleic acid-based techniques: a systematic review and meta-analysis. Inflamm Bowel Dis 14:401-10.
    This analysis of 49 studies of PCR and 6 studies of DNA in situ hybridization concluded that "there is an association between MAP and CD, across many sites, by many investigators, and controlling for a number of factors; however, this association remains controversial."

13. Juste RA, Elguezabal N, Garrido JM, Pavon A, Geijo MV, et al. (2008) On the prevalence of M. avium subspecies paratuberculosis DNA in the blood of healthy individuals and patients with inflammatory bowel disease. PLoS ONE 2008 3:e2537.
    IS900 MAP DNA was identified using a nested PCR primer on the buffy coat of blood. MAP DNA was found in 47% of the healthy controls but only 16% of the IBD patients (both Crohn's and UC,

not differentiated in the results section). The authors concluded that the use of anti-MAP drugs "was responsible for the decreased prevalence of MAP DNA in patients with IBD."

14. Hermon-Taylor J, Barnes N, Clarke C, Finlayson C (1998) Mycobacterium paratuberculosis cervical lymphadenitis, followed five years later by terminal ileitis similar to Crohn's disease. BMJ 316: 449-453.

15. Thayer WR Jr, Coutu JA, Chiodini RJ, Van Kruiningen HJ, Merkal RS (1984) Possible role of mycobacteria in inflammatory bowel disease. II. Mycobacterial antibodies in Crohn's disease. Dig Dis Sci 29: 1080-1085.
    In this ELISA study, 23% of the Crohn's patients had positive elevated antibody titers for the MAP antigen versus none of the UC patients or healthy controls.

16. Nakase H, Nishio A, Tamaki H, Matsuura M, Asada M, et al. (2006) Specific antibodies against recombinant protein of insertion element 900 of Mycobacterium avium subspecies paratuberculosis in Japanese patients with Crohn's disease. Inflamm Bowel Dis 12: 62-69.
    Almost half of the Crohn's patients had antibodies to the recombinant IS900 protein in this ELISA study, in contrast to none of the UC patients or controls.

17. Polymeros D, Bogdanos DP, Day R, Arioli D, Vergani D, et al. (2006) Does cross-reactivity between mycobacterium avium paratuberculosis and human intestinal antigens characterize Crohn's disease? Gastroenterology 131: 85-96.
    In this ELISA study, while the most reactive self/MAP peptide pair was found in only 30% of Crohn's patients, reactivity against at least one MAP peptide was found in 86% of Crohn's patients, versus only 20% of the UC patients or controls. The authors focus on the possibility that MAP antigens could be a target of cross reactive immunity, rather than on their results: the vast majority of Crohn's patients have at least one antibody to MAP antigens in their bloodstreams.

18. Romero C, Hamdi A, Valentine JF, Naser SA (2005) Evaluation of surgical tissue from patients with Crohn's disease for the presence of Mycobacterium avium subspecies paratuberculosis DNA by in situ hybridization and nested polymerase chain reaction. Inflamm Bowel Dis 11: 116-125.
    As discussed in the text, the DNA in situ hybridization studies utilizing labeled IS900 probes show positive results in a high percentage of samples, but only small numbers of organisms (1-3) per sample. See this article, and [19-21].
    In this study, using a florescent labeled IS900 probe, 67% (8/12) of the Crohn's patients had positive in situ hybridization results. The exact number of organisms identified in each sample and the exact site where they were located were not stated. Ten of the 12 Crohn's samples and both of the ulcerative colitis samples were also positive for MAP DNA by PCR.

19. Sechi LA, Mura M, Tanda F, Lissia A, Solinas A, et al. (2001) Identification of Mycobacterium avium subsp. paratuberculosis in biopsy specimens from patients with Crohn's disease identified by in situ hybridization. J Clin Microbiol 39: 4514-4517.
    This study and [20] utilized a "biotinylated" IS900 probe that had been amplified by PCR prior to labeling. 73% of the Crohn's biopsies had at least one sample with positive results, with granulomas or granulomas like cells containing positive staining. From the figures, one to three organisms/granuloma were present. All of the ulcerative colitis and non-IBD controls were negative.

20. Sechi LA, Mura M, Tanda E, Lissia A, Fadda G, et al. (2004) Mycobacterium avium sub. paratuberculosis in tissue samples of Crohn's disease patients. New Microbiol 27: 75-77.

Almost 70% of the tested Crohn's resection samples showed positive signals, but which part of the bowel wall had these positive signals was not reported. All of the ulcerative colitis and non-IBD controls were negative, as in their study [19] of biopsy specimens.

21. Hulten K, El-Zimaity HM, Karttunen TJ, Almashhrawi A, Schwartz MR, et al. (2001) Detection of Mycobacterium avium subspecies paratuberculosis in Crohn's diseased tissues by in situ hybridization. Am J Gastroenterol 96: 1529-1535.

This DNA in situ hybridization study utilized a digoxigenin labeled IS900 probe. 6 of the 15 patients with Crohn's disease and granulomas had positive signals. The positive signals were in myofibroblasts and macrophages in the lamina propria, however, not within the granulomas. Two of the 21 ulcerative colitis patients also had positive signals, presumably in the same locations. Biopsy and resection specimens were reported together.

22. Jeyanathan M, Boutros-Tadros O, Radhi J, Semret M, Bitton A, et al. (2007) Visualization of Mycobacterium avium in Crohn's tissue by oil-immersion microscopy. Microbes Infect 9:1567-73.

Traditional histochemical stains have historically been negative. Only very recently has this single study shown M. avium complex organisms (of which MAP is a subspecies) in tissue sections by traditional histochemical stains. Jeyanathan and colleagues successfully demonstrate that part of the reason MAP hasn't been directly visualized in histologic sections of tissue is that we haven't looked hard enough. Their direct visualization required 1000 magnification (x100 objective) rather than the standard 400 magnification (x40 objective). This study identified single M. avium organisms (i.e., small number of organisms) in 59% (10/17) of the Crohn's patients and 40% of the UC patients using Ziehl Neelsen staining and Fite staining (a Ziehl Neelsen variant). The organisms they identified were in the submucosa, but not otherwise located (for example, in blood vessels, lymph vessels, etc.).

23. De Hertogh G, Aerssens J, Geboes KP, Geboes K (2008) Evidence for the involvement of infectious agents in the pathogenesis of Crohn's disease. World J Gastroenterol 14: 845-52.

"Numerous other specific bacteria have been proposed as candidate causative agents of Crohn's disease, including Pseudomonas maltophilia, Mycobacterium kansasii, Chlamydia trachomatis, Bacteroides fragilis and Listeria monocytogenes…"

24. Hermon-Taylor J, Bull T (2002) Crohn's disease caused by Mycobacterium avium subspecies paratuberculosis: a public health tragedy whose resolution is long overdue. J Med Microbiol 51:3-6.

25. Greenstein RJ (2003) Is Crohn's disease caused by a mycobacterium? Comparisons with leprosy, tuberculosis, and Johne's disease. Lancet Infect Dis 3: 507-514.

"Viable MAP has been isolated and subsequently cultured by some laboratories from some patients with Crohn's disease…this finding may be considered to satisfy the first and second postulates...

Cultured human MAP has been administered orally to goats. Intestinal and mesenteric inflammation compatible with early Johne's disease was identified in one study of a single animal…In a more extensive study (of four animals)…the organisms was identified in mesenteric lymph nodes….MAP have been inoculated intravenously or intraperitoneally in several species

resulting in liver and splenic granulomata in normal mice and splenic isolates in immune- deficient mice…These data may be interpreted as confirming Koch's third postulate.
MAP of human and bovine origin has been re-isolated and recultured. These data may be interpreted as confirming Koch's fourth postulate."

Note for the second reference [25] on page 1 of the PDF; "…and municipal tap water…" Greenstein writes: "MAP is found in the potable water supply of large industrialized nations. Mycobacteria are at least two orders of magnitude more resistant to chlorine purification than Escherichia coli. MAP survives higher concentrations of chlorine (two parts per million) than the 1.1 parts per million routinely achieved with first-use municipal water in the USA. Additionally, mycobacteria are more resistant to chlorine purification at the low nutrient, low temperature, and increased-pH conditions that may be encountered in water systems."

26. Chamberlin W, Borody T, Naser S (2007) MAP-associated Crohn's disease MAP, Koch's postulates, causality and Crohn's disease. Dig Liver Dis 39: 792-4.
Relman's criteria include the presence of the nucleic acid sequence belonging to the putative pathogen in most cases of the disease, and the concept of biologic plausibility, i.e., "the nature of the microorganism inferred from the available sequence should be consistent with the known biological characteristics of that group of organisms." This criterion of biologic plausibility is fulfilled most emphatically by MAP, which causes a chronic inflammation of the intestines in every species it infects.

27. Millar D, Ford J, Sanderson J, Withey S, Tizard M, et al. (1996) IS900 PCR to detect Mycobacterium paratuberculosis in retail supplies of whole pasteurized cows' milk in England and Wales. Appl Environ Microbiol 62: 3446-3452.
"Up to 25%" of samples of whole pasteurized cow's milk were positive for MAP by IS900 PCR during "peak periods in January to March and in September to November." Half of the IS900 positive samples were also culture positive.

28. Hruska K, Bartos M, Kralik P, Pavlik I (2005) Mycobacterium avium supsp. Paratuberculosis in powdered infant milk. Available: http://www.paratuberculosis.org/pubs/proc8/abst3b_k8.htm. Accessed 24 February 2009.
This article appeared only in abstract form in the 2005 proceedings of the International Colloquium on Paratuberculosis. "IS900 (MAP DNA) was found in 25 (49% of) samples originating from seven manufacturers from six different countries."

29. Naser SA, Schwartz D, Shafran I (2000) Isolation of Mycobacterium avium subsp paratuberculosis from breast milk of Crohn's disease patients. Am J Gastroenterol 95:1094-5.
IS900 PCR on centrifugal pellets from MGIT tubes inoculated with breast milk from 2 women with Crohn's disease was positive for the presence of MAP DNA.

30. Pickup RW, Rhodes G, Arnott S, Sidi-Boumedine K, Bull TJ, et al. (2005) Mycobacterium avium subsp. paratuberculosis in the catchment area and water of the River Taff in South Wales, United Kingdom, and its potential relationship to clustering of Crohn's disease cases in the city of Cardiff. Appl Environ Microbiol 71: 2130-2139.
This study found MAP in the water and soil sediment of a river running through the above mentioned city, and closely correlated the high incidence of Crohn's disease in most of the wards of the city with the direction of winds carrying aerosols from the river.

31. Whan L, Ball HJ, Grant IR, Rowe MT (2005) Occurrence of Mycobacterium avium subsp. paratuberculosis in untreated water in Northern Ireland. Appl Environ Microbiol 71: 7107-7112.

8% of the samples were positive for MAP by at least one of the methods used (PCR and two different types of culture mediums).

32. Pickup RW, Rhodes G, Bull TJ, Arnott S, Sidi-Boumedine K, et al. (2006) Mycobacterium avium subsp. paratuberculosis in lake catchments, in river water abstracted for domestic use, and in effluent from domestic sewage treatment works: diverse opportunities for environmental cycling and human exposure. Appl Environ Microbiol 72:4067-77.

33. Grewal SK, Rajeev S, Sreevatsan S, Michel FC Jr (2006) Persistence of Mycobacterium avium subsp. paratuberculosis and other zoonotic pathogens during simulated composting, manure packing, and liquid storage of dairy manure. Appl Environ Microbiol 72:565-74.

"Livestock manure from dairies is largely stored as a liquid in lagoons or as solid packed manure, and then periodically applied to agricultural lands. Liquid manure storage and application can potentially lead to a variety of adverse environmental effects primarily related to liquid runoff, leaching, and odors." This study of 3 methods of storing and treating manure (thermophilic composting, low temperature composting/packing or liquid lagoon storage) showed that MAP "persisted for more than 2 months" regardless of which treatment/storage method was used.

34. Collins MT (2003) Paratuberculosis: review of present knowledge. Acta Vet Scand 44:217-21.

Collins writes: "Water contaminated with manure from domesticated animals is another potential route of human exposure that has not been examined. Given that M. avium is commonly found in domestic (city) water supplies due to its inherent resistance to chlorine, it is rational to believe that M. paratuberculosis too could contaminate surface water that end(s) up in domestic water supplies and thus expose humans to this potential pathogen."

35. Podolsky DK (2002) Inflammatory bowel disease. N Engl J Med 347: 417-429.

Podolsky writes: "Inflammatory bowel disease is thought to result from inappropriate and ongoing activation of the mucosal immune system driven by the presence of normal luminal flora. This aberrant response is most likely facilitated by defects in both the barrier function of the intestinal epithelium and the mucosal immune system."

36. Jeyanathan M, Alexander DC, Turenne CY, Girard C, Behr MA (2006) Evaluation of in situ methods used to detect Mycobacterium avium subsp. paratuberculosis in samples from patients with Crohn's disease. J Clin Microbiol 2006 44:2942-50.

What is the difference between seeing small numbers of MAP organisms under the microscope, and seeing large numbers? What counts as finding "large numbers" of MAP organisms in Crohn's tissues? Jeyanathan and colleagues argue that pluribacillary infection results in a bacterial burden that allows identification of the organism at x400 magnification (40x objective) rather than oil immersion (x1000 magnification, with the 100x objective). They write:

"Examination of ZN (Ziehl-Neelsen) stained sections of samples from paucibacillary murine infections revealed that individual mycobacteria could only be visualized by careful examination under x 1,000 oil immersion. Occasional aggregates of several mycobacteria were noted and could be detected under magnification x 400, consistent with our experience with sections of multibacillary

Johne's disease, where aggregates of mycobacteria are readily visualized under magnification x 400."

Note for reference [36], page 2 of the PDF; "…only small numbers of organisms are present in the histologic lesions." Jeyanathan and colleagues write that Crohn's disease is "a human disease that is defined by the absence of detectable pathogens…"

37. Sartor RB (2005) Does Mycobacterium avium subspecies paratuberculosis cause Crohn's disease? Gut 54: 896-8.

38. El-Zaatari FA, Osato MS, Graham DY (2001) Etiology of Crohn's disease: the role of Mycobacterium avium paratuberculosis. Trends Mol Med 7:247-52.

39. Cheng VC, Yew WW, Yuen KY (2005) Molecular diagnostics in tuberculosis. Eur J Clin Microbiol Infect Dis 24:711-20.

40. Britton WJ, Lockwood DN (2004) Leprosy. Lancet 2004 363:1209-19.

41. Clarke CJ (1997) The pathology and pathogenesis of paratuberculosis in ruminants and other species. J Comp Pathol 116: 217-261.

42. Balfour Sartor R (2007) Bacteria in Crohn's disease: mechanisms of inflammation and therapeutic implications. J Clin Gastroenterol 41: 37-43.
Balfour Sartor argues that defects in epithelial barrier function allows nonspecific "injurious commensal" bacteria through the epithelium, rather than a specific pathogenic bacterium.

43. Van Kruiningen HJ, Colombel JF (2008) The forgotten role of lymphangitis in Crohn's disease. Gut 57: 1-4.

44. Hadfield H (1939) The primary histological lesion of regional ileitis. Lancet 2: 773-775.

45. Warren S, Sommers SC (1948) Cicatrizing Enteritis (Regional Ileitis) as a Pathologic Entity. Am J Pathology 24: 475-501.
Note for reference [45], page 6 of the PDF: "The early histopathologic studies of 'regional ileitis' …" Warren and Sommers write: "An identical sequence of changes (proliferating endothelial cells blocking lymphatics and transforming into giant cell granulomas) is found in the <u>*mesenteric*</u> *lymph channels of the affected segment and in its regional lymph nodes (emphasis added)."*

46. Pedica F, Ligorio C, Tonelli P, Bartolini S, Baccarini P (2008) Lymphangiogenesis in Crohn's disease: an immunohistochemical study using monoclonal antibody D2-40. Virchows Arch 452: 57-63.

47. Hatoum OA, Binion DG (2005) The vasculature and inflammatory bowel disease: contribution to pathogenesis and clinical pathology. Inflamm Bowel Dis 11: 304-313.

48. Binion DG, West GA, Volk EE, Drazba JA, Ziats NP, et al. (1998) Acquired increase in leucocyte binding by intestinal microvascular endothelium in inflammatory bowel disease. Lancet 352: 1742-1746.

49. Hatoum OA, Binion DG, Otterson MF, Gutterman DD (2003) Acquired microvascular dysfunction in inflammatory bowel disease: Loss of nitric oxide-mediated vasodilation. Gastroenterology 125: 58-69.

50. Sankey EA, Dhillon AP, Anthony A, Wakefield AJ, Sim R, et al. (1993) Early mucosal changes in Crohn's disease. Gut 34: 375-381.

51. Danese S, Sans M, de la Motte C, Graziani C, West G, et al. (2006) Angiogenesis as a novel component of inflammatory bowel disease pathogenesis. Gastroenterology 130: 2060-2073.

52. Pousa ID, Maté J, Gisbert JP (2008) Angiogenesis in inflammatory bowel disease. Eur J Clin Invest 38: 73-81.

53. Wakefield AJ, Sawyerr AM, Dhillon AP, Pittilo RM, Rowles PM, et al. (1989) Pathogenesis of Crohn's disease: multifocal gastrointestinal infarction. Lancet 2: 1057-1062.

54. Geller SA, Cohen A (1983) Arterial inflammatory-cell infiltration in Crohn's disease. Arch Pathol Lab Med 107: 473-475.

55. Van Patter WN, Bargen JA, Dockerty MB, Feldman WH, Mayo CW, et al. (1954) Regional enteritis. Gastroenterology 26: 347-450.

56. Matson AP, Van Kruiningen HJ, West AB, Cartun RW, Colombel JF, et al. (1995) The relationship of granulomas to blood vessels in intestinal Crohn's disease. Mod Pathol 8: 680-685.

57. Tiwari A, VanLeeuwen JA, McKenna SL, Keefe GP, Barkema HW (2006) Johne's disease in Canada Part I: clinical symptoms, pathophysiology, diagnosis, and prevalence in dairy herds. Can Vet J 47: 874-882.

58. Nacy C, Buckley M (2008) Mycobacterium Avium Paratuberculosis: Infrequent Human Pathogen or Public Health Threat? A Report from the American Academy of Microbiology. Available: http://academy.asm.org/index.php?option=com_content&task=view&id=56&Itemid=53. Accessed 24 February 2009

59. Mehta PK, Karls RK, White EH, Ades EW, Quinn FD (2006) Entry and intracellular replication of Mycobacterium tuberculosis in cultured human microvascular endothelial cells. Microb Pathog 41: 119-124.

Mehta and colleagues write in their introduction: "Invasion and intracellular replication in various endothelial cell lines and in mouse lung endothelium by bacterial pathogens such as Streptococcus pneumoniae, Chlamydia pneumoniae, Staphylococcus aureus, Haemophilus influenza and Porphyromonas gingivalis have been well documented. It has also been hypothesized that antibiotic

treatment of infected endothelial cells could fail to eradicate the intracellular pathogens resulting in the infected cells potentially serving as a reservoir for chronic or persistent infections."

60. Scollard DM, McCormick G, Allen JL (1999) Localization of Mycobacterium leprae to endothelial cells of epineurial and perineurial blood vessels and lymphatics. Am J Pathol 154: 1611-1620.

61. Stappenbeck TS, Hooper LV, Gordon JI (2002) Developmental regulation of intestinal angiogenesis by indigenous microbes via Paneth cells. Proc Natl Acad Sci U S A 99: 15451-15455.

62. Cane G, Moal VL, Pagès G, Servin AL, Hofman P, et al. (2007) Up-Regulation of Intestinal Vascular Endothelial Growth Factor by Afa/Dr Diffusely Adhering Escherichia coli. PLoS ONE 2: e1359.
Cane and colleagues argue that the "enteroadherent, pro-inflammatory" E. Coli strain they investigated promotes angiogenesis by increasing the production of vascular endothelial growth factor.

63. Wormser GP (2007) Discovery of new infectious diseases - bartonella species. N Engl J Med 356: 2346-2347.
Wormser writes: "A unique facet of infection with bartonella is the ability of these microorganisms to stimulate neovascular proliferation in tissues, presumably by causing endothelial-cell proliferation and migration." But can't any organism that can cause a fistula cause neovascular proliferation, i.e., granulation tissue? See the discussion in the article of the possible role of angiogenesis in fistula formation.

64. del Gaudio A, Bragaglia RB, Boschi L, del Gaudio GA, Accorsi D (1997) A new approach in the management of Crohn's disease: observations in 20 consecutive cases. Hepatogastroenterology 44: 1095-1103.

65. Lorenzo GA, Poticha SM, Beal JM (1972) Mesenteric lymphatics in regional enteritis. Arch Surg 105: 375-378.

66. Ferrante M, Penninckx F, De Hertogh G, Geboes K, D'Hoore A, et al. (2006) Protein-losing enteropathy in Crohn's disease. Acta Gastroenterol Belg 69: 384-389.

67. Kalima TV, Saloniemi H, Rahko T (1976) Experimental regional enteritis in pigs. Scand J Gastroenterol 11: 353-362.

68. Tonelli P (2001) Il Linfedema deli'Intestino (malattia di crohn). Napoli: Global Press S.R.L.

69. Lakatos PL, Szamosi T, Lakatos L (2007) Smoking in inflammatory bowel diseases: good, bad or ugly? World J Gastroenterol 13: 6134-6139.

70. Wakefield AJ, Sawyerr AM, Hudson M, Dhillon AP, Pounder RE (1991) Smoking, the oral contraceptive pill, and Crohn's disease. Dig Dis Sci 36: 1147-1150.
Wakefield and colleagues discuss how cigarette smoke might enhance the "focal activation of intravascular coagulation" present in "intramural" vessels supplying segments of intestine affected

by Crohn's disease" by "direct endothelial cell injury" that causes "impaired prostacyclin production" by and a "decreased fibrinolytic capacity" of the injured endothelial cells.

71. Cotran RS, Kumar V, Robbins SL (1994) Robbins Pathologic Basis of Disease, 5th Edition. Philadelphia: W.B. Saunders Company. 1400 p.
The features of both Crohn's disease and transmural bowel infarction include the sharply defined demarcation of the affected bowel wall from the normal adjacent bowel, and the "edematous, thickened and rubbery" nature of the affected bowel wall. See pages 788 and 801.

Note for reference [71], p 7 of the PDF: "Granulation tissue is physiologic angiogenesis." Cotran and colleagues (p. 79) specifically make the link between granulation tissue and angiogenesis. They write: "The term granulation tissue derives from its pink, soft, granular appearance on the surface of wounds, but it is the histologic features that are characteristic: the proliferation of new small blood vessels and fibroblasts. New vessels originate by budding or sprouting of pre-existing vessels, a process called angiogenesis or neovascularization."

72. Anthony A, Dhillon AP, Pounder RE, Wakefield AJ (1997) Ulceration of the ileum in Crohn's disease: correlation with vascular anatomy. J Clin Pathol 50: 1013-1017.
Both mesenteric and antimesenteric vasa recta arteries might have large numbers of MAP organisms, but the mucosal ulcers and inflammation have a predilection for the mesenteric border because of the differences between the submucosal plexuses supplied by the long and short vasa recta, discussed in the article.

73. Anthony A, Pounder RE, Dhillon AP, Wakefield AJ (2000) Similarities between ileal Crohn's disease and indomethacin experimental jejunal ulcers in the rat. Aliment Pharmacol Ther 14: 241-245.

74. Anthony A, Dhillon AP, Pounder RE, Wakefield AJ (1999) The colonic mesenteric margin is most susceptible to injury in an experimental model of colonic ulceration. Aliment Pharmacol Ther 13: 531-535.

75. Rappaport H, Burgoyne FH, Smetana HF (1951) The pathology of regional enteritis. Mil Surg 109: 463-502.
Their "obliterative" endarteritis is a synonym for proliferative endarteritis. See [Figure 3]. By definition, obliterative endarteritis is a narrowing or obstruction of the lumen of a small artery by the proliferation of the intimal layer, i.e., the proliferation of the endothelial cells constituting that intimal layer.

76. Knutson H, Lunderquist A, Lunderquist A (1968) Vascular changes in Crohn's disease. Am J Roentgenol 103: 380-385.

77. Brahme F, Lindström C (1970) A comparative radiographic and pathological study of intestinal vaso-architecture in Crohn's disease and in ulcerative colitis. Gut 11: 928-940.

78. Kalima TV, Peltokallio P, Myllärniemi H (1975) Vascular pattern in ileal Crohn's disease. Ann Clin Res 7: 23-31.

79. Brahme F, Hildell J (1976) Angiography in Crohn's disease revisited. AJR Am J Roentgenol 126: 941-951.

80. Rosai J (1996) Ackerman's Surgical Pathology, 8th edition. St. Louis: Mosby. 2732 p.
"The lining of the (anal) fistula is made of granulation tissue…(p. 801)."

81. Bataille F, Klebl F, Rümmele P, Schroeder J, Farkas S, et al. (2004) Morphological characterisation of Crohn's disease fistulae. Gut 53: 1314-1321.
This is the only "detailed histological investigation of Crohn's fistulae" ever published. Both the Crohn's and non Crohn's fistulas "were lined by granulation tissue with conspicuous pale plump histiocytes and a dense network of tender capillaries." The cells in the Crohn's fistulas consisted of an inner (closer to the lumen or tract) layer of T lymphocytes, and an outer layer of B lymphocytes, in sharp contrast to the cells in the non Crohn's fistulas, which were macrophages. Are these T and B lymphocytes reacting, however ineffectively, to the intracellular MAP organisms in the "dense network of tender capillaries"?

Note for the second reference [81] on page 7 of the PDF: "…this mass of small blood vessels, is usually permanent." Bataille and colleagues write: "Permanent closure of fistulae can only be achieved in approximately 20 – 30% of patients."

82. National Cancer Institute. Understanding Cancer Series: Angiogenesis. http://www.cancer.gov/cancertopics/understandingcancer/angiogenesis. Accessed 24 February 2009.

83. Peyrin-Biroulet L, Chamaillard M, Gonzalez F, Beclin E, Decourcelle C, Antunes L, Gay J, Neut C, Colombel JF, Desreumaux P (2007) Mesenteric fat in Crohn's disease: a pathogenetic hallmark or an innocent bystander? Gut. 56: 577-583.

Note for the second reference [83] on page 7 of the PD; "…there is a paucity of literature devoted to the subject." Peyrin-Biroulet and colleagues write: "Of more than 6000 papers published in the last 20 years, less than 0.2% of them have mentioned the term adipose tissue." A June 2008 search of PubMed Central using the terms "creeping fat" and "Crohn's" revealed 12 references, out of a total for just "Crohn's" of 27, 927.

Note for the second reference [83] on page 8 of the PDF; "…there is actually a hyperplasia, an increase in number, of the individual adipocytes that comprise the mesentery." Peyrin-Biroulet and colleagues write: "Furthermore, visceral adipocytes are significantly smaller, resulting in a fourfold increased number of adipocytes throughout the mesentery of patients with Crohn's disease compared to controls." They conclude that "(t)aken together, these observations indicate that mesenteric obesity is a common and specific feature of Crohn's disease and may be due to hyperplasia rather than hypertrophy of the mesenteric adipocytes."

84. Sheehan AL, Warren BF, Gear MW, Shepherd NA (1992) Fat-wrapping in Crohn's disease: pathological basis and relevance to surgical practice. Br J Surg 79: 955-958.
Sheehan and colleagues write: "The pathologic features of 225 small intestinal resections were reviewed and fat-wrapping was seen only in Crohn's disease."

85. Greenstein RJ, Collins MT(2004) Emerging pathogens: is Mycobacterium avium subspecies paratuberculosis zoonotic? Lancet 364:396-397.

86. Charrière G, Cousin B, Arnaud E, André M, Bacou F, et al. (2003) Preadipocyte conversion to macrophage. Evidence of plasticity. J Biol Chem 278: 9850-9855.
Charrière and colleagues write: "Analysis of the literature revealed that adipocytes and monocytes/macrophage lineages have many features in common. In particular, proteins or functions

known to be specific to one lineage are characterized in the other…preadipocytes and adipocytes secrete numerous inflammatory cytokines such as tumor necrosis factor-α and are sensitive to lipopolysaccharide activation.”

87. Desruisseaux MS, Nagajyothi, Trujillo ME, Tanowitz HB, Scherer PE (2007) Adipocyte, adipose tissue, and infectious disease. Infect Immun 75: 1066-1078.
Desruisseaux and colleagues write: “…the adipocyte proper serves as an important target for the intracellular parasite Trypanosoma cruzi, the cause of Chagas' disease. In chronic Chagas' disease, adipocytes may represent an important long-term reservoir for parasites from which relapse of infection can occur.” Do mesenteric adipocytes serve a similar reservoir function for MAP in Crohn's disease?

88. Neyrolles O, Hernández-Pando R, Pietri-Rouxel F, Fornès P, Tailleux L, et al. (2006) Is adipose tissue a place for Mycobacterium tuberculosis persistence? PLoS ONE 1: e43.
These authors demonstrated non-replicating M. tuberculosis in mature adipocytes from a variety of sites taken from autopsies; the mesenteries were not sampled.

89. Yamamoto K, Kiyohara T, Murayama Y, Kihara S, Okamoto Y, et al. (2005) Production of adiponectin, an anti-inflammatory protein, in mesenteric adipose tissue in Crohn's disease. Gut 54(6):789-96.
Yamamoto and colleagues write: “Quantitative analyses indicated that the size of adipocytes in hypertrophied mesenteric adipose tissue of CD patients…was approximated one quarter the size of those in mesenteric adipose tissue from controls….On the other hand, the number of adipocytes per unit area in hypertrophied mesenteric adipose tissue of CD patients was 3.5-fold that of adipocytes in the mesenteric adipose tissue from controls.” That is, there was hyperplasia of the adipocytes.

90. Crohn BB, Ginzburg L, Oppenheimer GD (1932) Regional ileitis. A pathological and clinical entity. JAMA 99: 1323-1329.
Crohn and colleagues write: The “giant cells…could be demonstrated frequently in all layers of the intestine.” In the acute non-resected cases seen on the operating table, “(t)he mesentery of the terminal ileum is greatly thickened and contains numerous hyperplastic glands.” Lymph nodes were called glands in these early descriptions. Note the description of the nodes as hyperplastic.

91. Lockhart-Mummery HE, Morson BC (1960) Crohn's disease (regional enteritis) of the large intestine and its distinction from ulcerative colitis. Gut 1: 87-105.
Lockhart-Mummery and Morson write: “In most of the cases of Crohn's disease of the colon the granulomatous reaction includes non-caseating giant-cell systems which are distributed through all the layers of the bowel wall, as well as in the regional lymph nodes.”

92. Chiodini RJ (1989) Crohn's Disease and the mycobacterioses: a review and comparison of two disease entities. Clinical Microbiol Rev 2: 90-117.

93. Behr MA, Semret M, Poon A, Schurr E (2004) Crohn's disease, mycobacteria, and NOD2. Lancet Infect Dis 4: 136-7.

94. Liu Y, van Kruiningen HJ, West AB, Cargun RW, Cortet A, et al. (1995) Immunocytochemical evidence of Listeria, Escherichia coli, and Streptococcus antigens in Crohn's disease. Gastroenterology 108: 1396-404.

In this study, for example, “mesenteric lymph nodes” were examined for “Bacteroides vulgatus, Borrelia burgdorferi, Escherichia coli, Listeria monocytogenes, Streptococcus spp., bovine viral diarrhea virus, influenza A virus, measles virus, parainfluenza virus, and respiratory syncytial virus” but not for MAP.

95. Wakefield AJ, Sankey EA, Dhillon AP, Sawyerr AM, More L, et al. (1991) Granulomatous vasculitis in Crohn's disease. Gastroenterology 100: 1279-1287.

96. Mooney EE, Walker J, Hourihane DO (1995) Relation of granulomas to lymphatic vessels in Crohn's disease. J Clin Pathol 48: 335-338.

97. Ryan P, Bennett MW, Aarons S, Lee G, Collins JK, et al. (2002) PCR detection of Mycobacterium paratuberculosis in Crohn's disease granulomas isolated by laser capture microdissection. Gut 665-670.

98. Greenstein RJ, Su L, Haroutunian V, Shahidi A, Brown ST (2007) On the action of methotrexate and 6-mercaptopurine on M. avium subspecies paratuberculosis. PLoS ONE 2: e161.

99. Greenstein RJ, Su L, Shahidi A, Brown ST (2007) On the action of 5-amino-salicylic acid and sulfapyridine on M. avium including subspecies paratuberculosis. PLoS ONE 2: e516.

100. Greenstein RJ, Su L, Juste RA, Brown ST (2008) On the action of cyclosporine A, rapamycin and tacrolimus on M. avium including subspecies paratuberculosis. PLoS ONE 3: e2496.

101. Domingue GJ Sr, Woody HB (1997) Bacterial persistence and expression of disease. Clin Microbiol Rev 10: 320-44.

102. Chiodini RJ, Van Kruiningen HJ, Thayer WR, Coutu JA (1986) Spheroplastic phase of mycobacteria isolated from patients with Crohn's disease. J Clin Microbiol 24: 357-63.

103. Kobayashi K, Blaser MJ, Brown WR (1989) Immunohistochemical examination for mycobacteria in intestinal tissues from patients with Crohn’s disease. Gastroenterology 96: 1009-1015.

Immunohistochemical examination for MAP in Crohn’s tissues has, like traditional histochemistry, also been negative. This study tested antibodies to MAP strain Linda (a strain isolated from a human), M. tuberculosis, and the common mycobacterial antigen liparabinomannan and “did not detect mycobacteria in any of the 67 (surgical resection) specimens from 30 patients examined.”

104. Relman DA, Schmidt TM, MacDermott RP, Falkow S (1992) Identification of the uncultured bacillus of Whipple's disease. N Engl J Med 327:293-301.

105. Wear DJ, Margileth AM, Hadfield TL, Fischer GW, Schlagel CJ, et al. (1983) Cat scratch disease: a bacterial infection. Science 221:1403-5.

106. Warren JR, Marshall B (1983) Unidentified curved bacilli in the stomach of patients with gastritis and peptic ulceration. Lancet 1(8390):1311-5.

107. Sherris JC, Ryan KJ, Ray CG, Plorde JJ, Corey L, Spizizen (1984) Medical Microbiology: An Introduction to Infectious Diseases (New York; Elsevier Publishing Co, 1984) 694 p.

108. Marshall BJ, Armstrong JA, McGechie DB, Glancy RJ (1985) Attempt to fulfill Koch’s postulates for pyloric campylobacter. Med J Aust 142: 436 – 439.
